# Dental Student Perspectives of a Comprehensive-Based Teaching Methodology: A Confidence, Effectiveness, and Challenge Report

**DOI:** 10.1155/2020/8842008

**Published:** 2020-08-28

**Authors:** Susan Hattar, Abeer AlHadidi, Sandra Altarawneh, Suha Abu-Ghazaleh, Mohammad Hammad

**Affiliations:** ^1^Department of Conservative Dentistry, School of Dentistry, The University of Jordan, Amman, Jordan; ^2^Department of Oral Maxillofacial Surgery, Oral Medicine and Periodontology, School of Dentistry, The University of Jordan, Amman, Jordan; ^3^Department of Removable Prosthodontics, School of Dentistry, The University of Jordan, Amman, Jordan; ^4^Department of Pediatric Dentistry and Orthodontics, School of Dentistry, The University of Jordan, Amman, Jordan

## Abstract

*Background*/*Objectives*. The holistic methodology in education has been widely appreciated and implicated in dental schools in the last decade. Our department of conservative dentistry decided to reform the educational model of teaching from a traditional requirement-based model to a hybrid model incorporating comprehensive care treatment. The aim of our study was to assess students' confidence and perspectives regarding the benefits of a comprehensive model of teaching. *Materials and Methods*. A questionnaire was distributed at the end of the academic year 2018-2019 and designed to investigate students' opinions on the benefits of the new model of teaching, as well as difficulties encountered and possible shortcomings. In addition, self-perceived confidence level was assessed for the purpose of comparing confidence during supervised tasks versus confidence during comprehensive patient care. *Results*. Complete responses were gathered from 127 students out of 202, giving a response rate of 63%. The majority of students believed that the comprehensive model of teaching allowed them to better address patients' needs, gave higher satisfaction, positively influenced self-confidence, permitted greater exposure to clinical techniques, and enhanced reasoning and analytical skills. However, their confidence was still lower in comprehensive patient management when compared to supervised tasks. *Conclusion*. Our students showed an appreciation of the comprehensive care model. Self-learning and didactic skills were enhanced. It would, therefore, be beneficial to adapt this methodology to earlier years and other disciplines to enhance the effectiveness of education and achievement of learning outcomes.

## 1. Introduction

Academic dental institutions vary in their pedagogical systems from traditional methods in which students are asked to complete a certain number of tasks to more interactive methods such as case-based learning [1–3], competency-based curricula [4], group discussions, e-modules, and comprehensive dental care [5]. Furthermore, trends such as outreach teaching experiences and community dental care have proven to be useful adjuncts to meet the increasing demand for well-trained graduates [6]. All these new educational systems have shown to enhance achievement, acquisition of knowledge, and student professional development [7–9].

In the last decade, comprehensive educational approaches have received great attention in both medical and dental academic fields [10]. Most dental schools have felt the need and importance to change their model of teaching from a subject-specific approach to a more holistic approach that results in an evidence-based quality of oral health care. From one view, it enables students to look upon the greater image of treating patients and not to focus on one precise problem. Furthermore, patients' needs are met, and health care is provided in a proper sequential manner which is not applicable when a “student-centered approach” is practiced [9–11].

Our faculty has been recognized by the Association for Dental Education in Europe (ADEE) to have European standards of dental education with a curriculum in agreement with EU guidelines. The feedback from the committee stressed on the idea of having both vertical and horizontal integration. After revisiting our dental curriculum, opinions started shifting toward adopting a more holistic way of thinking for our students. In our institution, these prevailing philosophies could not have been applied in their pure form due to restraints of large number of students. Nevertheless, changes were introduced by incorporating comprehensive dental care in our restorative curriculum. The departmental curriculum change mainly involves the fifth year students who, in addition to their competencies, are required to treat at least one patient requiring complex dental restorative work. The treatment involves multidisciplines and must be conducted in a sequential structured manner. At the end of the year, students present their clinical work and discuss the treatment plan and management steps in front of a jury. In theory, dental schools are no longer required to provide a total learning environment [12], so we contemplated that these changes would encourage students to practice self-assessment and self-learning resulting in a positive educational outcome. Nevertheless, from our belief that certain skills are necessary to be attained to a competent level, a mix between varied philosophies of teaching was adopted.

Dental educators are faced with challenges related to the evolution and continuous change inherent to the profession. Among others, new technologies emerged, such as CAD CAM technology [13], computerized casts [14], biomimetic materials [15], lasers [16], and genetic research [17]. Most dental schools follow methods of assessment that are mainly competency-based [18]. In general, their learning outcomes are achieved by students completing a number of basic tasks unassisted and to a competent level. At our dental school, the curriculum of restorative dentistry is divided into preclinical training at the third year and clinical training at the level of the fourth and fifth year. Conforming with general educational guidelines, we have a staff/student ratio of 1 : 8 for the clinical training years. In each clinical session, staff members from endodontic and conservative subdisciplines are responsible of supervising and guiding students throughout their clinical steps. Previously, our clinical courses were solely requirement-based, where students had a designated number of tasks to finish prior to graduation. A few years ago, we started implementing the comprehensive-based learning in accordance with the reforms in dental education seen worldwide. The aim was to enhance our dental education and to produce practitioners who are able to think independently and critically while managing patients' problems and adapting to future changes. Our system might be considered a hybrid system, since students are still asked to accomplish certain individualized tasks and achieve competency in specific procedures. In the novel setting, students will be encouraged not only to develop in their clinical and intellectual skills but also to become independent, self-directed, life-long learners.

When evaluating the success of any dental program, stakeholders at different levels, including students [5] and staff, as well as patients must be provided with the ultimate benefit of this program [19]. Furthermore, the intended learning outcomes must be met. The aim of this study was to investigate the students' opinions on the benefits and challenges of a comprehensive case-based course and give us a better understanding of how they viewed and valued the experience. Additionally, the study will provide us insight into student-perceived confidence levels in different clinical settings, supervised compared to unsupervised contexts. These findings will enable us to address shortcomings of the curriculum and help us establish new reforms to improve both structure and didactic organization for the purpose of graduating competent and confident dentists.

## 2. Materials and Methods

### 2.1. Data Collection

At the end of the academic year 2018-2019, all fifth year DDS students (*N* = 202) were invited to complete a course evaluation E-questionnaire (Qualtrics®) before their final examinations. The data represented students' self-reported achieved level of confidence since the questionnaire was delivered at the end of the year. The questionnaire was anonymous, and students were informed verbally about the questionnaire to optimize the number of respondents.

The questionnaire consisted of different parts. The first section investigated the general level of student confidence in supervised tasks, compared to the confidence level when treating patients comprehensively. Students' confidence was reported on a five-point scale (1 = extremely not confident, 2 = not confident, 3 = somehow confident, 4 = confident, and 5 = extremely confident).

The second section aimed to obtain a perspective of students' experience and attitudes toward the implication of the comprehensive-based restorative curriculum. Multiple key points such as benefits, skills acquired, and work stress were addressed, and students' responses were rated on a five-point Likert scale (5 = strongly agree, 4 = somewhat agree, 3 = neither agree nor disagree, 2 = somewhat disagree, and 1 = strongly disagree) as seen in similar studies [1].

Finally, students were asked about difficulties encountered during the year that could have affected their progress, engagement, or achievement of intended learning outcomes of the course, such as lack of adequate time or patient- and staff-related issues.

### 2.2. Data Analysis

Validation of the questionnaire was performed by asking 10 of the students to complete the questionnaire prior to the distribution. Data were recorded using the statistical package for social sciences SPSS Statistics 23 (IBM; Armonk, NY) (SPSS v14). A Pearson chi-squared test was used for comparison, and *p* < 0.05 was set as a statistically significant level.

### 2.3. Ethical Approval and Consent to Participate

This study was reviewed and approved by the Institutional Review Board/Deanship of Scientific Research of the University of Jordan (Ref # 18/2020/49 and 19/2020/49). The introduction of the questionnaire defined the purpose and objectives of the study. The authors also stated clearly that participation is completely voluntary with no penalties associated with refusal or withdrawal from participation. Consent was implied by responding to the questionnaire.

## 3. Results

One hundred and twenty-seven students responded to the questionnaire, giving a response rate of 63% of which 76% were females. All participants answered the questions with no records of missing data.

### 3.1. Perceived Confidence in Comprehensive Dental Care Compared to Supervised Tasks

In both clinical settings, more than half of the students reported being either extremely confident or confident in performing their tasks (supervised 83%, comprehensive patient care 58%).  Table 1 shows that the students' perceived confidence level was lower when performing comprehensive dental care compared to their confidence when performing completely supervised clinical procedures (*p* < 0.05).

On the other hand, the number of students reporting being somehow confident seemed significantly higher in comprehensive dental care compared to supervised tasks (*p* < 0.05).

### 3.2. Perception and Attitudes toward the Comprehensive-Based Learning Experience

Students were asked about their views of the comprehensive-based model, and the impact of it beholds on their satisfaction, confidence, acquired skills/knowledge, perceived stress, and patients' needs, as shown in Table 2.

**Figure 1:**
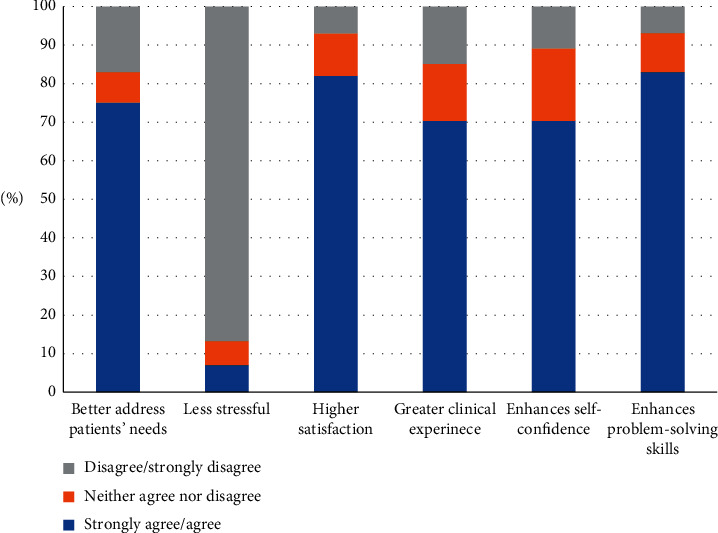
Percentages of students' responses regarding benefits of the comprehensive patient care.

To better understand the variation in the students' responses to the preference questions, the responses were collapsed into three categories by grouping the numbers of students who reported strongly agree with agree and disagree with strongly disagree.  Figure 1 shows that 75% of our students agreed that the comprehensive-based experience allowed them to better address patients' needs. Furthermore, they reported that it enhanced both their didactic (84%) and clinical skills (71%). The majority also stated that it gave them higher satisfaction (82%) and enhanced their self-confidence (71%). On the other hand, 87% of the students seemed to view this experience as being a stressful experience.

### 3.3. Students' Reported Challenges

Students were asked to treat their patients in a proper sequential timely manner while documenting each step and performing follow-up after completion of the treatment. When they were asked about the difficulties they encountered during the various stages of treatment, 74% reported lack of time as a handicap, 57% reported problems with patient drop out, and 41% mentioned problems related to staff supervision.

## 4. Discussion

The holistic approach in dental education has multiple advantages. Through this philosophy of teaching, all aspects associated with disease are taken into account when diagnosing and implementing treatment. This is one reason why this approach has been universally embraced. On the other hand, this pathway allows students to gain management skills and decision-making aptitudes in a problem-based learning approach. Another strength point is the student's exposure to real life situations that they will encounter in their future career [18]. This methodology has also been shown to increase student confidence in clinical reasoning, problem solving, and creativeness [7], which reflects positively on their performance and relationship with their patients [20].

From another aspect, competencies designed by experts are necessary skills that represent the backbone to guide the development of the curriculum content, student assessment, and accreditation. Similarly, competency-based education has been suggested to improve critical thinking and autonomy while embracing knowledge and confidence as well [8]. Nevertheless, the holistic approach in dental education cannot always be practiced in its full conception due to inherent institutional constraints. Our current curriculum may be described as a hybrid one, since it includes competencies related to operative, endodontic, and fixed prosthodontic subspecialties, as well as comprehensive care treatments performed in a multidisciplinary line.

For such an approach to succeed, all aspects of the educational environment must be well contemplated and properly planned. In order to identify the difficulties associated with the implication of this model, we gathered students' input for the purpose of adopting improvement methodologies. As primary stakeholders, their perception of the quality of education and feedback is valuable [21]. When asked about the struggles encountered, the students referred to patients' commitment, time and staff-related issues as impeding the true benefit of comprehensive-based education. From one view, the contributing role of teaching staff is of utmost importance for the success of educational programs [22]; however, the variability in educational experience and teaching methods [23] might have afflicted the aspired result. To overcome this matter, we set regular rotations to establish some sense of consistency and to allow students to be exposed to different learning experiences [11]. A true calibration of educators must also be provided, so that students can make the best out of this experience. Furthermore, assigning mentors to a small group of students might prove efficient for the purpose of sustainability of treatment planning and student achievement as has been shown by other studies [11]. For the second statement regarding lack of time, this might be overcome by reducing the number of secondary competencies and allowing the students to designate more time and effort for the comprehensive diagnosis, evaluation, and treatment of their patients. One study at the University of Bergen revealed that the holistic approach did not improve student satisfaction with teaching [10]. Their student comments were related to the teaching staff numbers and commitment, as well as lack of calibration regarding evaluation strategies.

A true appreciation and judgement of this methodology could be expected, since the student sample who answered the questionnaire was exposed to both educational models. In the fourth year, their curriculum consisted of a competency and requirement-based course. In the fifth year, in addition to the former, students were asked to perform comprehensive dental treatments, which encompassed integrated disciplines such as periodontics, endodontics, and prosthodontics. Nevertheless, it would be speculative to assume that this experience resulted in a positive impact on students from every aspect. In our study, not all students seemed certain of the benefit of this teaching method. As simple as it may seem, selection of different clinical tasks is primordial for success. Research has linked multiple attributes to the effectiveness of clinical work such as relevance, realism, engaging, and possessing challenging topics [3]. The students' clinical work was widely diverse from simple operative work, single crown placement, or single rooted endodontic treatment, to more complex dental care with fixed bridges and multirooted endodontic treatments.

At the end of the academic year, when students were asked to present and discuss their clinical work, we noticed a perceptible improvement in their communication skills. Moreover, a majority of our students agreed that this approach resulted in more diverse clinical exposure and enhanced their intellectual skills. This positive impact on the achievements of intended learning outcomes was reinforced by the fact that students were motivated in every step of the treatment and spent time analyzing and perfecting their management. A similar survey performed at the University of Tennessee following a transition from a departmental model to a comprehensive model revealed that their students had a clear preference for the comprehensive care model, yet only half them thought that this model was less stressful [11], which was similar to our students' feedback.

It makes intuitive sense that students feel more confident in the tasks they have practiced frequently or when being supervised [24]. However, one question that is constantly raised is whether the perceived need for assistance is justified. Most of our students are accustomed to working in an instructor-based environment. Our results demonstrated that students felt less confidence during comprehensive dental management compared to their confidence during supervised tasks. This is somehow comprehendible since students probably sense great responsibility and a constant motif to achieve the highest standards of treatment. Moreover, they are asked to present their clinical work in front of a jury at the end of the year. Therefore, building up students' self-esteem should be one of the primordial outcomes to develop [25].

As witnessed in many other schools, the delivery of modern evidence-based approaches is not without challenges [26]. Nonetheless, dental schools should be active in reviewing and modifying their curricula [18]. We believe that traditional teaching methods are outdated and lacking attributes necessary for student development. Hence, our philosophy was to establish a new curriculum which encourages integrated learning in an environment where students feel that they belong [27]. Although our school is located in the capital, recruitment of the “right patient at the right time for the right student” is not always evident. Demographics and referral protocols with the augmenting number of students make it a difficult process. Nevertheless, within the capacity and feasibility of our institution, future trends in curricular change are warranted. For instance, regular case-related discussions to enrich students' critical thinking might form a useful adjunct. In addition, interdisciplinary education revolving around themes and not specific points would be highly advantageous.

One of the limitations of our study was that validation of the questionnaire was not performed; however, a pilot study was conducted prior to data collection to confirm that students understood the questions properly. Furthermore, our response rate was fairly acceptable noting that 37% of students did not complete the survey. This might be due to the fact that the questionnaire was distributed at the end of the year when students were busy preparing for their final exams. Nevertheless, a larger sample size may have strengthened the study or provided more specificity.

We acknowledge the fact that surveys are capable of measuring perception of benefit from students' aspect but are inadequate for assessing actual skill acquisition, and further studies related to student productivity when using this educational system are indispensable. Another restriction is that our study reported the students' perception of one discipline which was restorative dentistry. This approach once adopted in other dental disciplines might demonstrate different outcomes. Furthermore, our system was not a pure comprehensive model as it is more of a hybrid system where competencies and requirements still exist.

## 5. Conclusions

This study provided insight into the prospects of using a comprehensive care model of education. In general, students confirmed our hypothesis that the comprehensive model provides great benefits in terms of clinical experience and intellectual skills. These results showed the student satisfaction that would drive us to go further in this transition to a complete holistic approach and a true integration between disciplines. This should become our top priority in the future. One way to achieve this is to invest in resources needed to accomplish these changes and to develop staff skills in new technologies and evaluation strategies.

## Figures and Tables

**Table 1:** Students self-perceived confidence level in different clinical settings.

Clinical task	Extremely confident (%)	Confident (%)	Somehow confident (%)	Not confident (%)	Extremely not confident
Supervised tasks	33	50	15	2	0
Comprehensive dental care	12	46	36	6	0

**Table 2:** Views of students regarding the comprehensive care experience, level of stress, satisfaction, and confidence.

Criterion	Number of students				
	Strongly agree	Agree	Neither agree nor disagree	Disagree	Strongly disagree
Better address patients' chief complaint and general needs	43	52	10	13	9
Offers less work stress	3	6	8	39	71
Allows greater exposure to clinical techniques and better clinical experience	41	49	19	11	7
Enhances reasoning/analysis and problem-solving skills	44	62	13	6	2
Positively influence your confidence	39	51	24	6	7
Gives higher satisfaction	63	41	14	3	6

## Data Availability

The data used to support the findings of this study have been deposited in the Research Square with DOI: 10.21203/rs.3.rs-27348/v1.

## References

[1] Yiu C. K. Y., McGrath C., Bridges S. (2012). Self-perceived preparedness for dental practice amongst graduates of the University of Hong Kong’s integrated PBL dental curriculum. *European Journal of Dental Education*.

[2] Fincham A. G., Shuler C. F. (2001). The changing face of dental education: the impact of PBL. *Journal of Dental Education*.

[3] Kim S., Phillips W. R., Pinsky L., Brock D., Phillips K., Keary J. (2006). A conceptual framework for developing teaching cases: a review and synthesis of the literature across disciplines. *Medical Education*.

[4] Whitney E. M., Walton J. N., Aleksejuniene J., Schönwetter D. J. (2015). Graduating dental students’ views of competency statements: importance, confidence, and time trends from 2008 to 2012. *Journal of Dental Education*.

[5] Divaris K., Barlow P. J., Chendea S. A. (2008). The academic environment: the students’ perspective. *European Journal of Dental Education*.

[6] Coe J. M., Brickhouse T. H., Bhatti B. A., Best A. M. (2018). Impact of community-based clinical training on dental students’ confidence in treating pediatric patients. *Journal of Dental Education*.

[7] McKenzie C. T. (2013). Dental student perceptions of case-based educational effectiveness. *Journal of Dental Education*.

[8] Yip H.-K., Smales R. J. (2000). Review of competency-based education in dentistry. *British Dental Journal*.

[9] Wu J., Feng X., Chen A., Zhang Y., Liu Q., Shao L. (2016). Comparing integrated and disciplinary clinical training patterns for dental interns: advantages, disadvantages, and effect on students’ self-confidence. *Journal of Dental Education*.

[10] Berge M. E., Berg E., Ingebrigtsen J. (2013). A critical appraisal of holistic teaching and its effects on dental student learning at University of Bergen, Norway. *Journal of Dental Education*.

[11] Dehghan M., Harrison J., Langham S., Scarbecz M., Amini M. (2015). Comparing comprehensive care and departmental clinical education models: students’ perceptions at the University of Tennessee College of Dentistry. *Journal of Dental Education*.

[12] Mossey P. (2004). The changing face of dental education. *British Dental Journal*.

[13] Zuskova L., Mortadi N. A. A., Williams R. J., Alzoubi K. H., Khabour O. F. (2019). Comparison of overall fit of milled and laser-sintered CAD/CAM crown copings. *International Journal of Dentistry*.

[14] Sfondrini M. F., Gandini P., Malfatto M., Di Corato F., Trovati F., Scribante A. (2018). Computerized casts for orthodontic purpose using powder-free intraoral scanners: accuracy, execution time, and patient feedback. *BioMed Research International*.

[15] Scribante A., Dermenaki Farahani M. R., Marino G. (2020). Biomimetic effect of nano-hydroxyapatite in demineralized enamel before orthodontic Bonding of Brackets and attachments: visual, adhesion strength, and hardness in in vitro tests. *BioMed Research International*.

[16] Al-Maliky M. A., Frentzen M., Meister J. (2019). Artificial caries resistance in enamel after topical fluoride treatment and 445 nm laser irradiation. *BioMed Research International*.

[17] Sabi S. H., Khabour O. F., Alzoubi K. H., Cobb C. O., Eissenberg T. (2020). Changes at global and site-specific DNA methylation of MLH1 gene promoter induced by waterpipe smoking in blood lymphocytes and oral epithelial cells. *Inhalation Toxicology*.

[18] Haden N. K., Hendricson W. D., Kassebaum D. K. (2010). Curriculum change in dental education, 2003–2009. *Journal of Dental Education*.

[19] Bissell V., Robertson D. P., McCurry C. W., McAleer J. P. G. (2018). Evaluating major curriculum change: the effect on student confidence. *British Dental Journal*.

[20] Fine P., Leung A., Bentall C., Louca C. (2019). The impact of confidence on clinical dental practice. *European Journal of Dental Education*.

[21] Henzi D., Davis E., Jasinevicius R., Hendricson W. (2007). In the students’ own words: what are the strengths and weaknesses of the dental school curriculum?. *Journal of Dental Education*.

[22] Shoaib L. A., Safii S. H., Naimie Z., Ahmad N. A., Sukumaran P., Yunus R. M. (2018). Dental students’ perceptions on the contribution and impact role of a clinical teacher. *European Journal of Dental Education*.

[23] Henzi D., Davis E., Jasinevicius R., Hendricson W. (2006). North American dental students’ perspectives about their clinical education. *Journal of Dental Education*.

[24] Sonbol H. N., Abu-Ghazaleh S. B., Al-Bitar Z. B. (2017). Undergraduate experience and self-assessed confidence in paediatric dentistry at the University of Jordan Dental School. *European Journal of Dental Education*.

[25] Abdulghani A. H., Almelhem M., Basmaih G. (2020). Does self-esteem lead to high achievement of the science college’s students? a study from the six health science colleges. *Saudi Journal of Biological Sciences*.

[26] Lynch C. D., Blum I. R., Wilson N. H. F. (2019). Leadership in dental education. *Journal of Dentistry*.

[27] Radford D. R., Hellyer P. (2016). Belongingness in undergraduate dental education. *British Dental Journal*.

